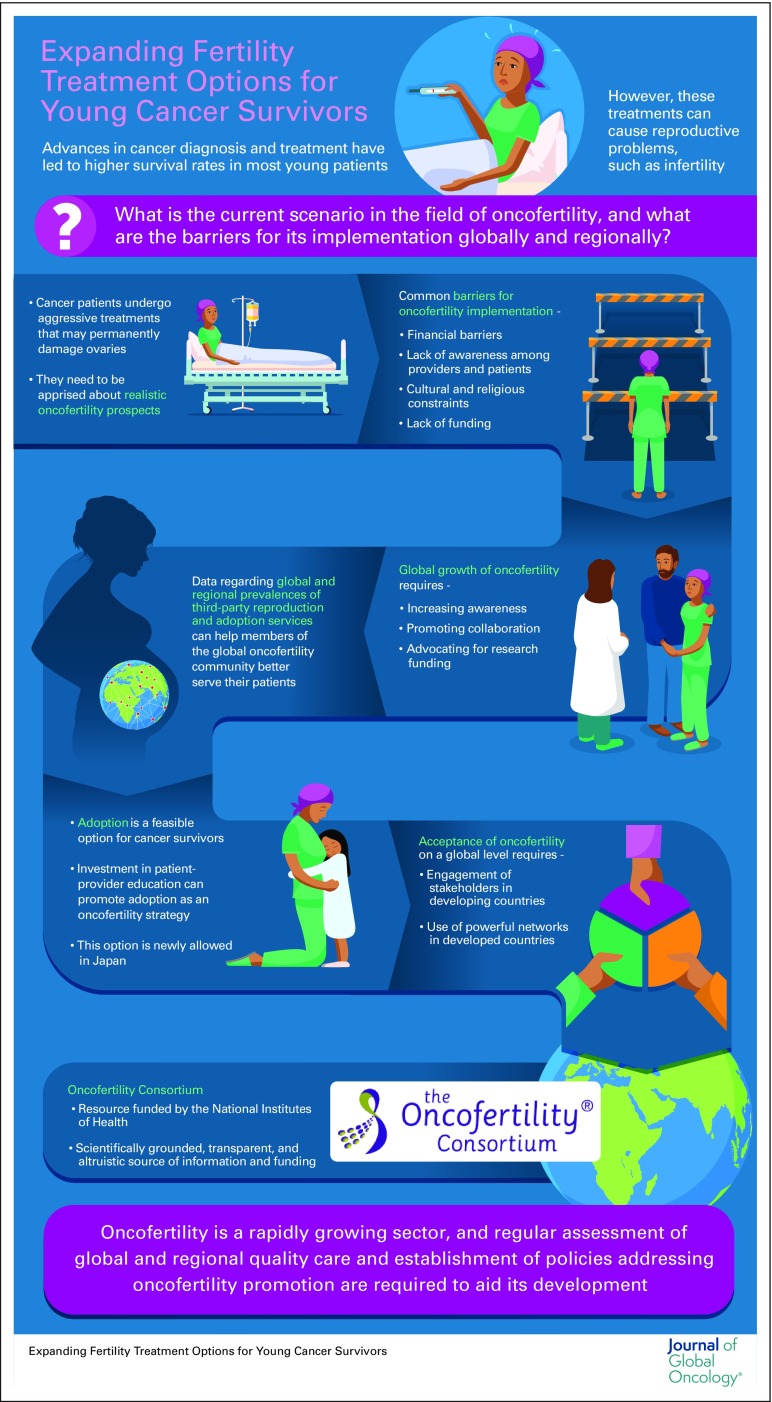# Oncofertility: A Global Perspective

**DOI:** 10.1200/JGO.20.00038

**Published:** 2020-03-02

**Figure:**